# Exploring T-cell exhaustion features in Acute myocardial infarction for a Novel Diagnostic model and new therapeutic targets by bio-informatics and machine learning

**DOI:** 10.1186/s12872-024-03907-x

**Published:** 2024-05-24

**Authors:** Nake Jin, Jiacheng Rong, Xudong Chen, Lei Huang, Hong Ma

**Affiliations:** 1 Department of Cardiology, The Second Affiliated Hospital, School of Medicine, Zhejiang University, State Key Laboratory of Transvascular Implantation Devices, Cardiovascular Key Laboratory of Zhejiang Province, Hangzhou, 310009 Zhejiang China; 2 Department of Cardiology, Ningbo Hangzhou Bay Hospital, Ningbo, 315300 Zhejiang China

**Keywords:** Myocardial infarction, T cell exhaustion, Immune cell, Bio-informatics, Machine learning, Diagnostic model, Therapeutic target

## Abstract

**Background:**

T-cell exhaustion (TEX), a condition characterized by impaired T-cell function, has been implicated in numerous pathological conditions, but its role in acute myocardial Infarction (AMI) remains largely unexplored. This research aims to identify and characterize all TEX-related genes for AMI diagnosis.

**Methods:**

By integrating gene expression profiles, differential expression analysis, gene set enrichment analysis, protein-protein interaction networks, and machine learning algorithms, we were able to decipher the molecular mechanisms underlying TEX and its significant association with AMI. In addition, we investigated the diagnostic validity of the leading TEX-related genes and their interactions with immune cell profiles. Different types of candidate small molecule compounds were ultimately matched with TEX-featured genes in the “DrugBank” database to serve as potential therapeutic medications for future TEX-AMI basic research.

**Results:**

We screened 1725 differentially expressed genes (DEGs) from 80 AMI samples and 71 control samples, identifying 39 differential TEX-related transcripts in total. Functional enrichment analysis identified potential biological functions and signaling pathways associated with the aforementioned genes. We constructed a TEX signature containing five hub genes with favorable prognostic performance using machine learning algorithms. In addition, the prognostic performance of the nomogram of these five hub genes was adequate (AUC between 0.815 and 0.995). Several dysregulated immune cells were also observed. Finally, six small molecule compounds which could be the future therapeutic for TEX in AMI were discovered.

**Conclusion:**

Five TEX diagnostic feature genes, CD48, CD247, FCER1G, TNFAIP3, and FCGRA, were screened in AMI. Combining these genes may aid in the early diagnosis and risk prediction of AMI, as well as the evaluation of immune cell infiltration and the discovery of new therapeutics.

**Supplementary Information:**

The online version contains supplementary material available at 10.1186/s12872-024-03907-x.

## Introduction

Acute myocardial infarction (AMI), also referred to as a heart attack, is a critical medical illness distinguished by the abrupt cessation of blood circulation to the cardiac muscle [1]. Although there have been notable breakthroughs in the detection and treatment of acute myocardial infarction (AMI), effectively managing this condition in a clinical setting continues to pose substantial challenges. The identification of reliable biomarkers and the explanation of underlying molecular mechanisms are essential for enhancing the diagnosis of acute myocardial infarction (AMI) and advancing the development of viable treatment approaches. T-cell exhaustion (TEX) refers to an immunological condition characterized by compromised T-cell activity, leading to diminished immune responses against tumors or viral infections [2]. Depleted T cells (T cells that undergo depletion) are functionally distinct from effector and memory T cells and are characterized by loss of effector function, increased and sustained expression of inhibitory receptors (IRS), altered epigenetic and transcriptional profiles, and altered metabolic patterns. Whether T cell depletion occurs is largely influenced by the level and number of inhibitory receptors (IRS) expressed, and immune checkpoint inhibitors can partially reverse the state of T cell depletion. In addition, the intensity of antigenic stimulation is an important factor. Pathways regarding T-cell depletion can be divided into three broad categories (1) intercellular signals (prolonged T-cell receptor engagement, co-stimulatory and/or co-inhibitory signals); (2) soluble cytokines (overproduction of inflammatory factors including IFN, IL-10, TGFβ-, etc.); (3) microenvironmental influences (driven by changes in the expression levels of chemokine receptors, adhesion molecules and trophic receptors). Microenvironmental effects may include altered tissue distribution and/or migration patterns and result in altered pathways for sensing oxygen tension (tumor suppressor and/or hypoxia-inducible factor pathways), pH, and nutrient levels. Tissue destruction and altered lymphoid organization may play a major role. Recent research has brought attention to the potential role of TEX in a range of pathological states, such as cancer and infectious illnesses [3, 4]. Nevertheless, the investigation of TEX’s role in acute myocardial infarction and its diagnostic implications remains limited in current research.

In the present context, we provide a comprehensive investigation with the objective of finding and describing genes associated to TEX for the purpose of diagnosing AMI. Our study aimed to elucidate the molecular pathways involved in TEX and its correlation with AMI by employing several analytical approaches, including the integration of gene expression profiles, differential expression analysis, gene set enrichment analysis, protein-protein interaction networks, and machine learning methods. In addition, we investigated the possible diagnostic significance of TEX-related genes and their relationships with immune cell profiles.

Previous research has focused on elucidating the molecular and cellular processes involved in AMI pathogenesis [5]. Studies have primarily investigated cardiac remodeling, inflammation, oxidative stress, and endothelial dysfunction as key contributors to AMI. While these studies have provided valuable insights, the role of TEX-related genes and their association with AMI remains relatively unexplored. To bridge this knowledge gap, we conducted an integrative analysis using publicly available gene expression datasets from patients with AMI and control samples. We performed differential expression analysis to identify significant TEX-related genes associated with AMI. Additionally, gene set enrichment analysis allowed us to unravel the functional annotations and pathways enriched in these TEX-related genes.

Furthermore, we have developed protein-protein interaction networks in order to investigate the intricate relationship between TEX-related genes and their associated interacting partners. The present analysis offers a comprehensive perspective on the molecular interactions that underlie thromboembolism (TEX) within the framework of AMI. In addition, machine learning methods were utilized to carefully choose a selection of diagnostic TEX genes. Subsequently, a score system was devised for the purpose of diagnosing AMI. Significantly, our findings were validated using different datasets, and the diagnostic effectiveness of the TEX-based scoring system was evaluated by receiver operating characteristic curve analysis. The introduction of a diagnostic nomogram facilitated the creation of a graphical depiction of the scoring system and its clinical ramifications. This study makes a valuable contribution to the expanding field of precision medicine by offering new perspectives on the molecular pathways that underlie thromboembolic events (TEX) in AMI. The potential development of targeted therapeutics and enhancement of AMI diagnosis can be facilitated with the identification of TEX-related genes linked to AMI and their interactions with immune cell profiles. In addition, the utilization of a diagnostic scoring system that relies on TEX-related genes has the potential to be a valuable asset in the process of risk classification and individualized treatment of patients with AMI.

To sum up, our integrated analysis provides a comprehensive understanding of TEX-related genes and their implications in AMI. The identification of diagnostic TEX genes and their interactions with immune cell profiles advances our understanding of AMI pathogenesis and offers potential targets for therapeutic intervention. Ultimately, our findings pave the way for future research in the development of personalized diagnostic and therapeutic strategies for AMI.

## Materials and methods

### Data source and preprocessing

Three datasets were downloaded on acute myocardial infarction from the NCBI Gene Expression Omnibus (GEO) database (https://www.ncbi.nlm.nih.gov/*)* [6]. The dataset information is as follows, and the raw data can be found in Sect. 0 - Raw Data:

A. **GSE66360**: This dataset includes 50 control samples and 49 samples from patients with acute myocardial infarction. The sequencing platform used is GPL570 [HG-U133_Plus_2] Affymetrix Human Genome U133 Plus 2.0 Array (blood is retained when the arterial sheath is placed in the cardiac catheter room after the onset of symptoms of chest pain to the emergency department to confirm the diagnosis of infarction according to the time requirements, and the specimen is processed within 36 h).
B. **GSE48060**: This dataset includes 21 control samples and 31 samples from patients with acute myocardial infarction. The sequencing platform used is GPL570 [HG-U133_Plus_2] Affymetrix Human Genome U133 Plus 2.0 Array (blood is retained within 48 h of diagnosis of heart attack.).
C. **GSE60993**: This dataset includes 7 control samples and 17 samples from patients with acute myocardial infarction. The sequencing platform used is GPL6884 Illumina HumanWG-6 v3.0 expression beadchip (blood is retained after confirming the diagnosis in the emergency department within 4 h of the onset of chest pain.).

The mRNA probe expression matrix file and the annotation file for each dataset were obtained by downloading the respective files associated with the sequencing platform. Subsequently, the probes were transformed into gene symbols, with the exclusion of any probes that did not correspond to a gene symbol. In instances where multiple probes were found to correspond to the same gene, we computed the mean value as the representative expression value for that particular gene. The aforementioned procedure yielded a gene expression matrix that can be utilized for subsequent investigation. Subsequently, the R package “sva” version 3.36.0 [7] was employed to mitigate batch effects in the expression profiles of datasets A and B. Subsequently, the datasets were pooled in order to generate a training set for subsequent analysis.

### Differential expression analysis

The differential analysis of acute myocardial infarction (AMI) compared to normal control groups was conducted using the “limma” package (version 3.34.7) in the R programming language [8]. The present study encompassed the computation of P-values and logFC (log-fold change) values for each individual gene. Furthermore, the Benjamini & Hochberg approach was utilized for the purpose of correcting for multiple testing [9], leading to the derivation of adjusted P-values (adj.P.Value). The differential expression was assessed using two metrics: fold change and significance. To identify genes that exhibit significant differential expression, we established a threshold of DEGs (|FC| ≥ 1.2 and adjusted P-values < 0.05) [10]. This threshold can also be stated as adjusted P-values < 0.05 and |log2FC| ≥ 0.263.

### Identification of differentially expressed genes associated with TEX

In order to discover genes associated with T-cell exhaustion (TEX), we initially obtained the TEX gene collection from existing scholarly publications [11]. Following that, we utilized the GSEA MsigDB database, available at the website (http://www.gsea-msigdb.org/gsea/downloads.jsp*)*, which is specifically designed for conducting gene set enrichment analysis (GSEA). Our objective was to procure the genes linked to four pertinent REACTOME pathways, namely TNF signaling, INTERLEUKIN-2 signaling, INTERFERON GAMMA signaling, and NATURAL KILLER CELL MEDIATED CYTOTOXICITY, from the REACTOME pathway database. Upon combining all the acquired genes and eliminating any instances of duplication, we have obtained a comprehensive collection of genes connected to TEX. In order to conduct a more comprehensive examination of the association between differentially expressed genes (DEGs) and TEX-related genes, we utilized an online tool (https://bioinfogp.cnb.csic.es/tools/venny/*)* to produce a Venn diagram. This enabled us to visually observe and identify the genes that are expressed at considerably different levels and are connected with the phenomenon of T-cell fatigue.

The resulting set of significant differentially expressed T-cell exhaustion-related genes can be used for the subsequent analysis. This comprehensive approach integrates information from literature, pathway databases, and statistical analysis, providing a very robust foundation for investigating the molecular mechanisms underlying T-cell exhaustion.

### Functional enrichment analysis of GO and KEGG pathways

In order to investigate the functional pathways and processes related to the overlapping genes identified in the aforementioned stage, we conducted Go and KEGG enrichment analyses utilizing the ‘clusterProfiler’ R package (version 4.0.5) [12]. The objective of this technique was to elucidate the primary functional annotations and pathways associated with these genes.

The Gene Ontology (GO) is a globally recognized framework for classifying gene functions. It offers a comprehensive collection of regulated vocabulary concepts that are used to define the characteristics of genes and gene products inside a biological organism [13]. The GO framework has three distinct ontologies, including molecular function, cellular component, and biological process, as outlined in reference [14]. In the present investigation, we employed the Benjamini & Hochberg method to compensate for multiple testing and derived the adjusted p-value, commonly referred to as adj.P.Value. To identify highly enriched Gene Ontology (GO) words and Kyoto Encyclopedia of Genes and Genomes (KEGG) pathways, we established a threshold of adjusted p-value (adj.P.Value) less than 0.05 and a count greater than 2.

For visualization and interpretation, we selected the most top 10 significant results based on adjusted p-values and presented them in a concise manner. This approach provides valuable insights into the functional annotations and pathways associated with the identified genes.

### Construction of interaction network

To explore the differential interactions between proteins associated with the TEX pathway, we utilized the STRING database to analyze (Version 11.0, http://string-db.org/*)* [15]. Subsequently, we constructed an interaction network to visualize the relationships among these gene products.

### Machine learning-based selection of diagnostic TEX genes

In this study, we utilized three machine learning methods, specifically LASSO [16], Random Forest (RF) [17], and SVM-RFE [18], in order to enhance the selection process of possible feature genes for diagnosing acute myocardial infarction (AMI). The LASSO approach, which is a technique for reducing dimensionality, shown its advantage over regression analysis in the evaluation of high-dimensional data. The LASSO study employed a regularization penalty parameter and the lmnet package for the purpose of selecting feature variables using a 10-fold cross-validation approach. The study employed the Random Forest algorithm, which is a supervised machine learning technique, to assess the ranking of acute myocardial infarction progression and TEX genes. This was achieved by implementing the Recursive Feature Elimination (RFE) method. The evaluation of prediction performance was conducted using ten-fold cross-validation, resulting in the identification of the top 30 genes with relative importance as the feature genes. The performance of SVM-RFE was superior to that of linear discriminant analysis and mean squared error approaches in the context of feature selection and redundancy elimination [19]. The identification of significant variables was achieved by the utilization of ten-fold cross-validation throughout the process of feature selection. The LASSO regression, Support Vector Machine (SVM), and Random Forest (RF) analyses were performed using the R packages “glmnet” [20], “e1071” [21], and “randomForest” [22], respectively. The genes that were discovered as the intersection among the three approaches were regarded as important TEX genes in the diagnosis of acute myocardial infarction (AMI). Ultimately, a diagnostic score formula was developed by the utilization of multiple logistic regression. This involved the calculation of regression coefficients (β) and gene expression levels (X) for each diagnostic TEX gene.

1 $${\bf{Diagnostic}}\,{\bf{score}} = 1 + {\rm{ (}}{{\rm{\beta }}_{\rm{1}}}{{\rm{X}}_{\rm{1}}}{\rm{ + }}{{\rm{\beta }}_{\rm{2}}}{{\rm{X}}_{\rm{2}}}{\rm{ + }}...{\rm{ + }}{{\rm{\beta }}_{\rm{n}}}{{\rm{X}}_{\rm{n}}}{\rm{)}}$$

#### Note

In this formula, β represents the regression coefficients, and X denotes the gene expression values.

### Validation and performance evaluation of diagnostic scoring

Using the Diagnostic score algorithm, we calculated the Diagnostic score values for each sample in both the training and validation sets. Afterwards, the Wilcoxon test was employed to compare the disparities in Diagnostic scores between the acute myocardial infarction and control groups. The R language’s “pROC” package (version 1.7.2) was utilized to generate Receiver Operating Characteristic (ROC) curves and compute the Area Under the Curve (AUC) as a means of assessing the diagnostic accuracy of TEX and the diagnostic score. The aforementioned analyses were performed on all datasets, encompassing both the training and validation sets.

### Visualization of diagnostic nomogram

The “rms” package (Version 5.1.2) was employed in the R programming language to develop a diagnostic nomogram for acute myocardial infarction (AMI) [23]. A nomogram can be described as a graphical representation of the regression equation, wherein a scoring system is developed by considering the magnitudes of regression coefficients associated with each independent variable [24]. A score is assigned to each value level of every independent variable, and subsequently, a cumulative score is computed for each sample. The likelihood of occurrence for each sample is calculated using a conversion function that relates the score to the probability of the outcome. The utilization of a calibration curve is employed as a means to evaluate the prediction capability of a nomogram. Ultimately, the clinical value was assessed through the utilization of decision curve analysis and clinical impact curve.

### Analysis of the correlation between TEX and immune cell profiles

The immune cell population present in a tissue exhibits a significant degree of diversity. Consequently, the examination of the immunological microenvironment or immune infiltration seeks to comprehend the specific makeup of immune cells within an afflicted tissue. In this investigation, the CIBERSORT (http://cibersort.stanford.edu/index.php*)* tool was utilized to determine the proportions of 22 immune cell subtypes. This was achieved by evaluating the expression levels of training dataset samples [25]. The CIBERSORT program employs linear support vector regression to deconvolute expression matrices of immune cell subtypes [26]. Subsequently, Wilcoxon tests were conducted to investigate the disparities in immune cell infiltration between samples of AMI and control. Additionally, the correlation between diagnostic TEX and immune cells was assessed.

### Gene set enrichment analysis

Gene Set Enrichment Analysis (GSEA) is a computer approach employed to evaluate the presence of statistically significant disparities between two biological states in a given set of genes [27]. The present work utilized Gene Set Enrichment Analysis (GSEA) to examine the enrichment of major Kyoto Encyclopedia of Genes and Genomes (KEGG) pathways in diagnostic TEX genes. A threshold was established to determine significance, with a P-value less than 0.05 and an absolute normalized enrichment score (NES) larger than 1 being considered as the cutoff criteria [28].

### Construction of protein-protein interaction (PPI) network for TEX diagnosis

We conducted an analysis of PPI using the GeneMANIA database (http://genemania.org/*).* The aim was to examine the association between diagnostic TEX genes and their 20 associated genes [29]. The objective of this investigation was to forecast the associations pertaining to co-localization, common protein domains, co-expression, and pathways.

### Prediction of candidate small molecule compounds

The “DrugBank” (https://go.drugbank.com/*)* is a web-based database that serves as a valuable resource for exploring the interactions between the selected genes and the small molecule compounds [30]. In our study, we utilized the DrugBank database to identify potential small molecule compounds that interact with the diagnostic TEX genes.

## Results

### Batch effect removal and data integration of transcriptomic profiling

**Fig. 1:**
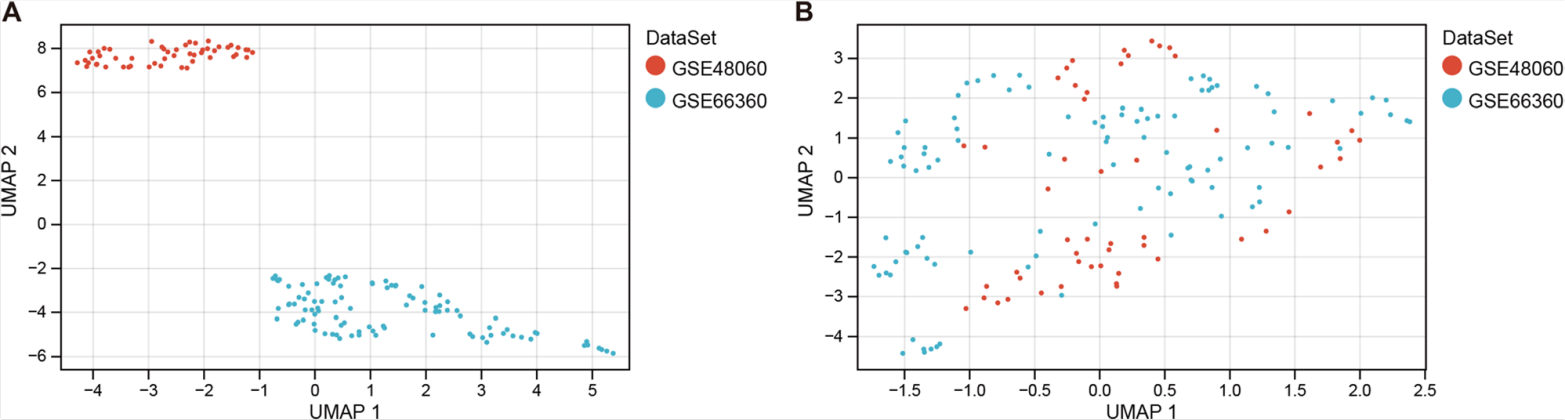
Sample Relationship Plot before (**A**) and after (**B**) batch effect removal using sva

Following the retrieval of the corresponding transcriptomic data, we employed the surrogate variable analysis (sva) algorithm to mitigate batch effects and integrate two distinct sets of the gene expression profiles into a unified dataset. Subsequently, a sample relationship plot was generated to visualize the interplay between the samples before and after removing the batch effects, as depicted in Fig. 1A and B. The resulting dataset consisted of 80 samples from patients with Acute Myocardial Infarction (AMI) and 71 control samples.

### Identification of differentially expressed genes (DEGs)

**Fig. 2:**
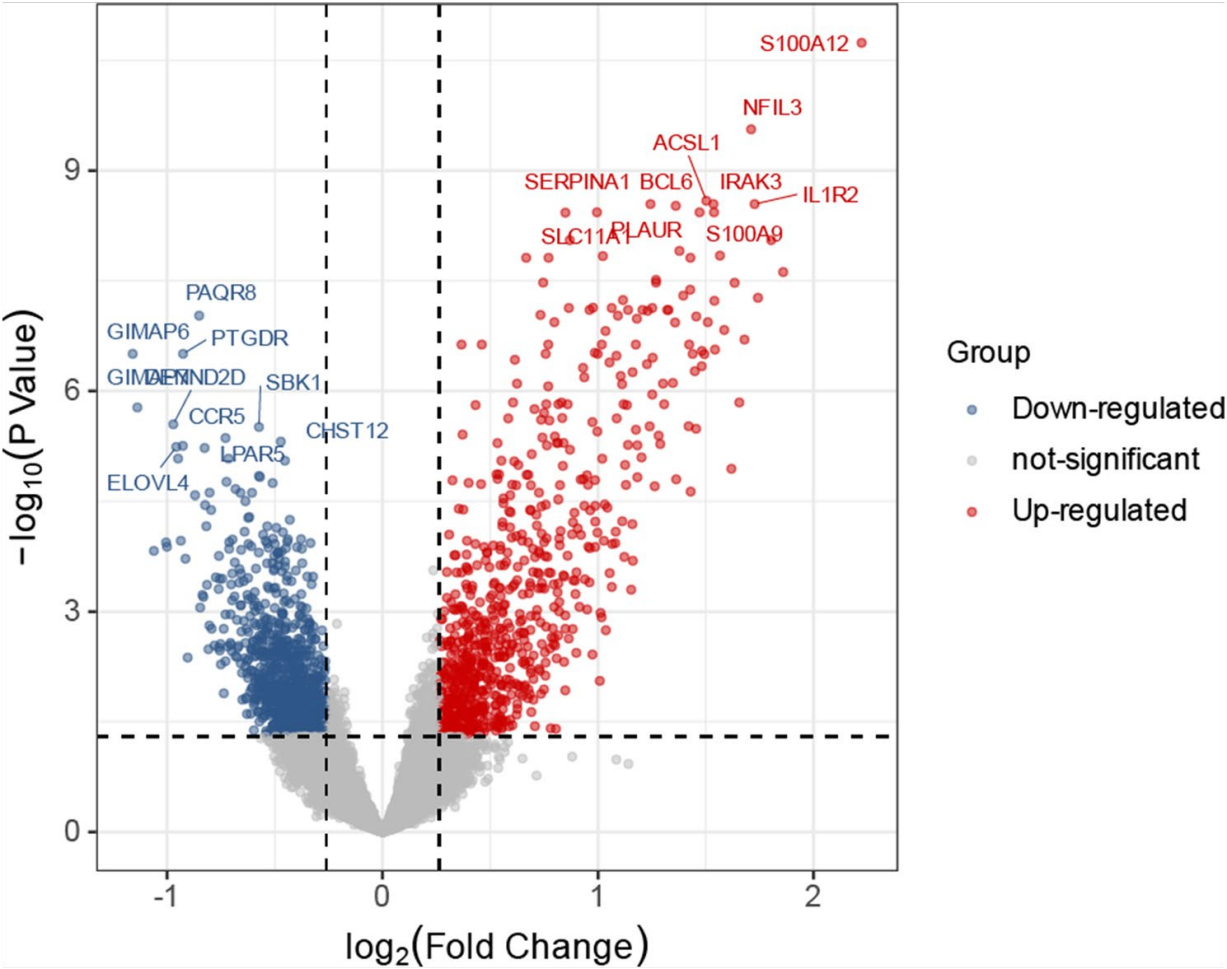
The volcano graphic illustrates the distribution of Differentially Expressed Genes (DEGs), with downregulated genes depicted in blue and upregulated genes depicted in red

Following the outlined methodology, we conducted a comparative analysis between AMI and control samples. The results, presented in Fig. 2 and documented in the supplementary Table 1, revealed a set of 1725 genes exhibiting differential expression.

### Identification of significantly differentially expressed genes related to T cell exhaustion

**Fig. 3:**
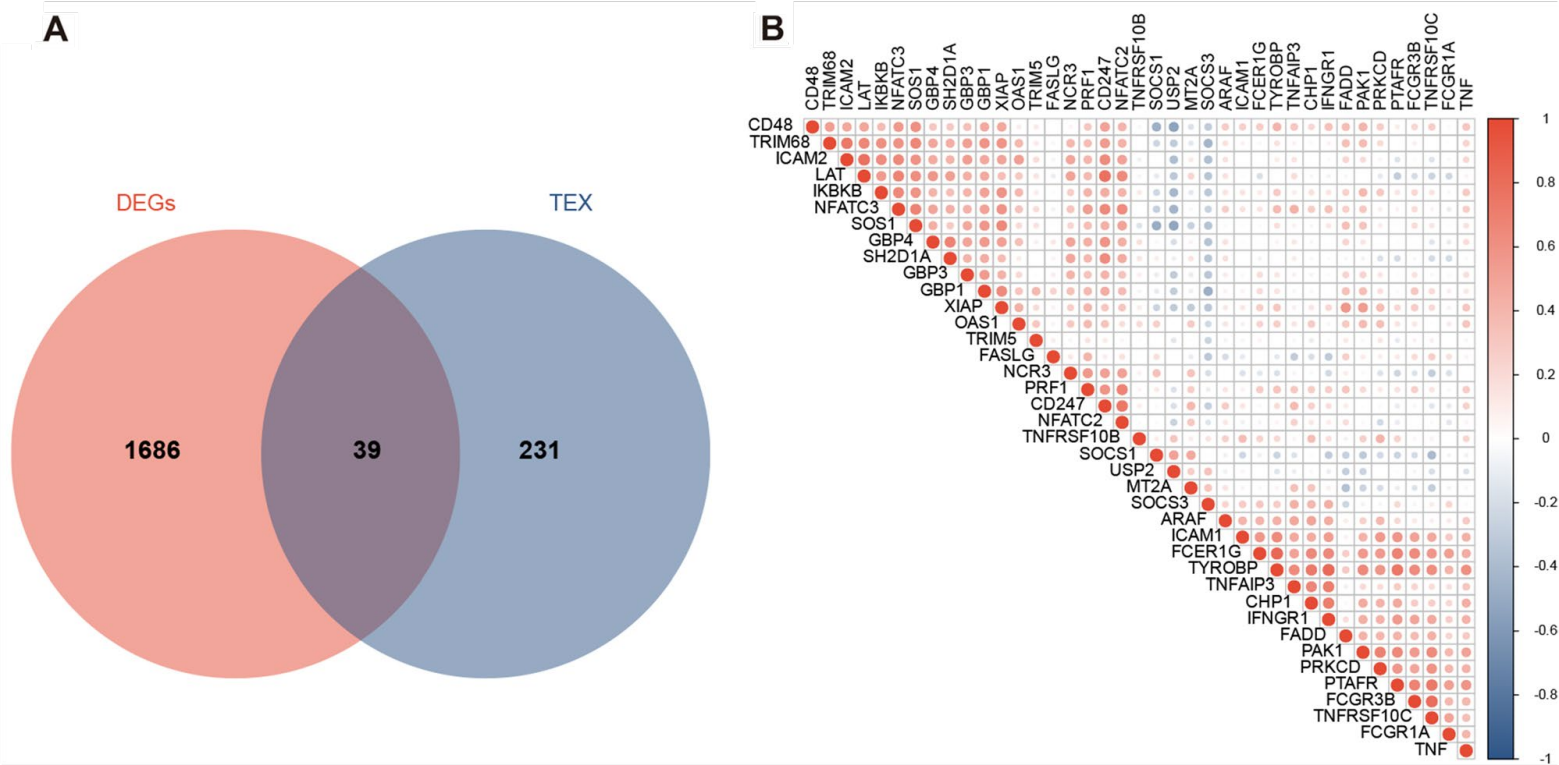
(**A**) Venn diagram depicting the intersection of T cell exhaustion-related gene results. (**B**) Heatmap showing the correlation analysis results

A total of 39 genes that are linked with T cell fatigue were discovered, as depicted in Fig. 3A. Following this, a correlation analysis was performed in order to investigate the expression patterns of the aforementioned 39 genes in samples of acute myocardial infarction (AMI). The findings are illustrated in Fig. 3B.

### Functional enrichment analysis of GO and KEGG pathways

**Fig. 4:**
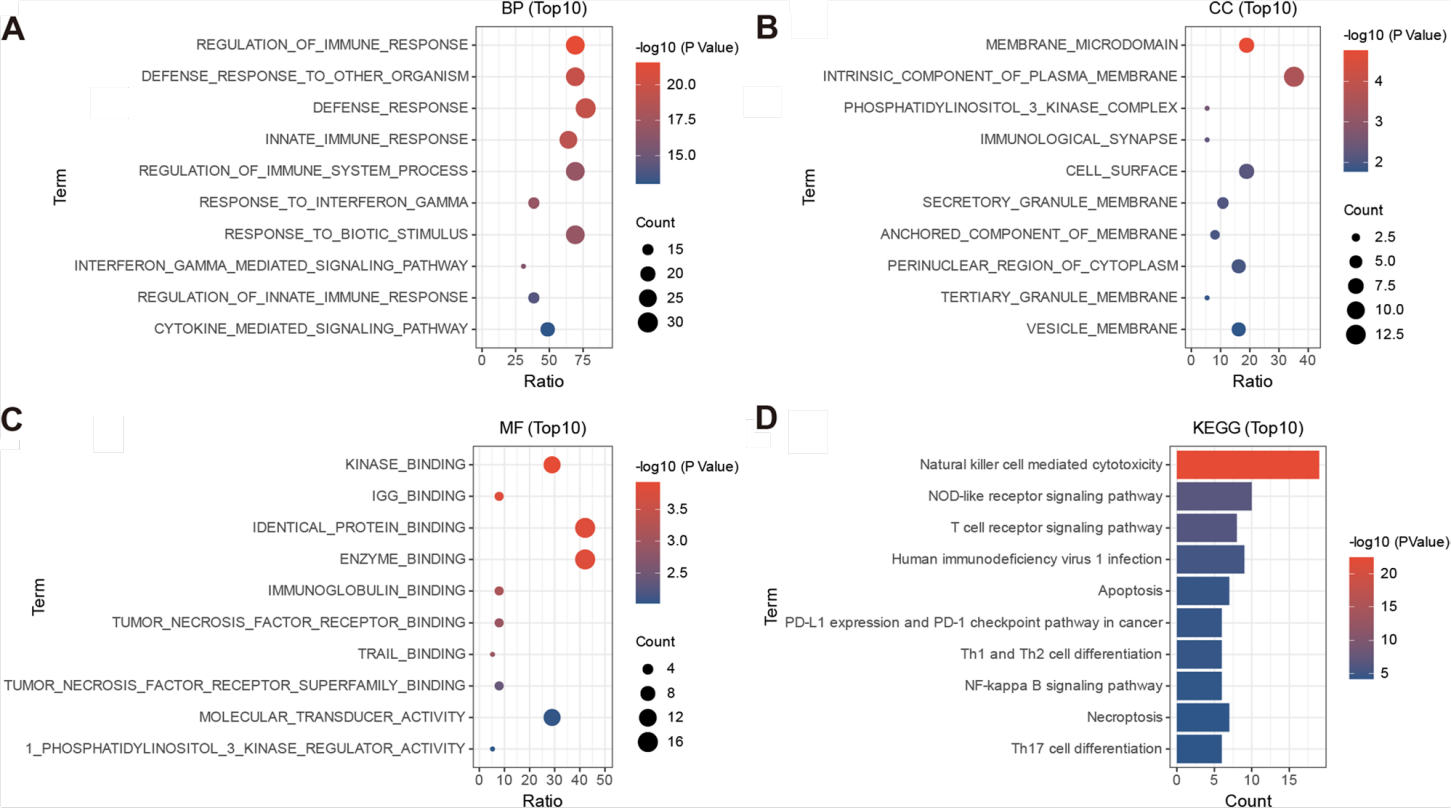
(**A**-**C**) Bubble plots representing the enrichment results. The horizontal axis represents the gene ratio, and the vertical axis represents the pathway names. The size of the bubbles indicates the number of genes, while the color intensity indicates the p-value. (**D**) Bar plot showing the pathway names on the vertical axis and the number of genes on the horizontal axis. The color intensity represents the p-value

Gene ontology (GO) functional analysis and KEGG pathway enrichment analysis were conducted on the aforementioned 39 intersecting genes in order to investigate the functional terms related with these pivotal genes. The enrichment results were derived using the adj.P values and are displayed in Fig. 4A-D. The top 10 results, sorted by p-value in ascending order, are shown.The thresholds for value, denoted as < 0.05, and count, denoted as ≥ 2, were established. A total of 478 biological processes (BP), 13 cellular components (CC), 18 molecular functions (MF), and 64 KEGG signaling pathways were identified as significantly enriched.

### Construction of the interaction network

**Fig. 5:**
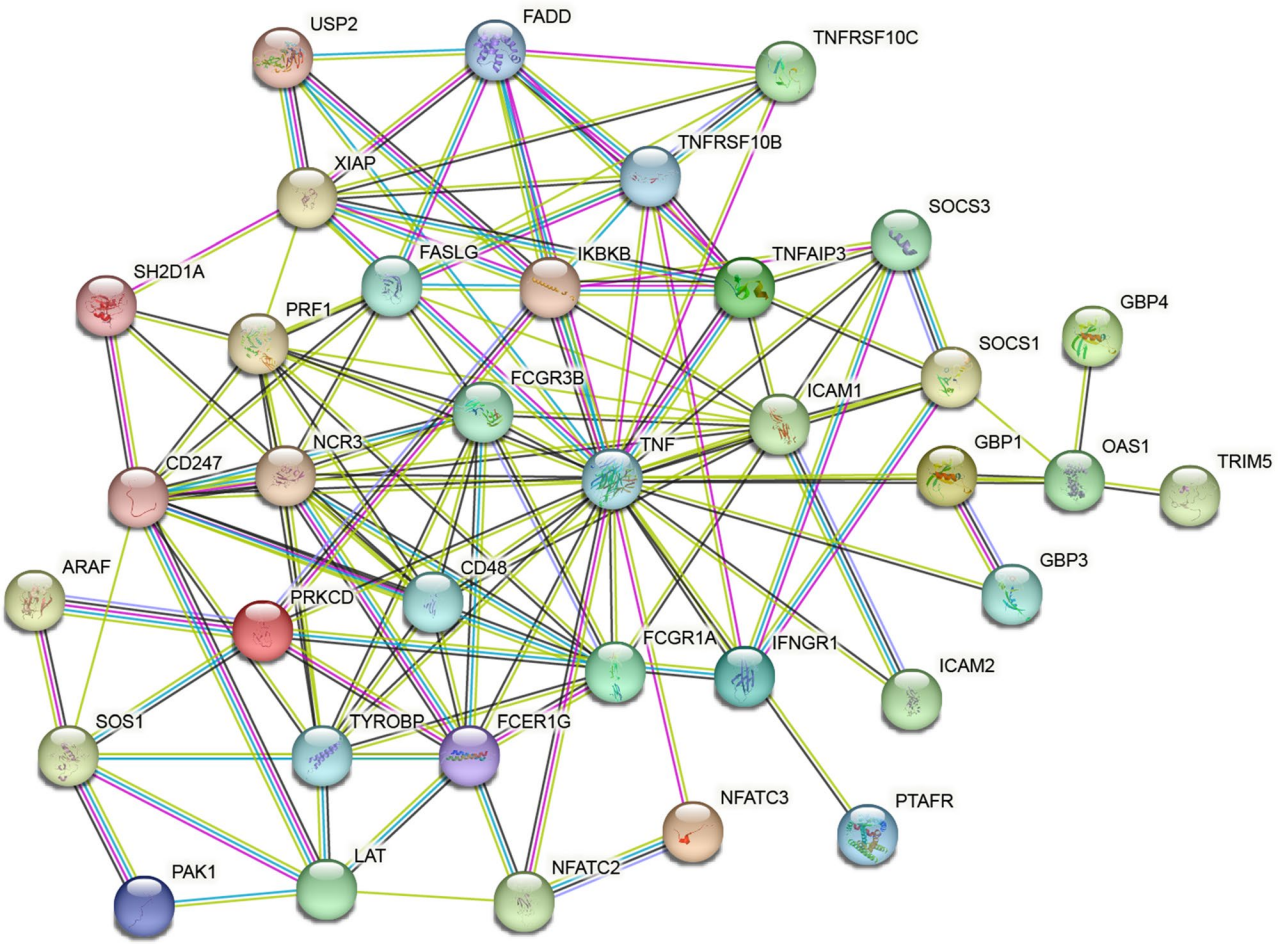
Protein-protein interaction network

We constructed an interaction network of TEX genes using the String database and Cytoscape software. The network, shown in Fig. 5, comprises 37 nodes and 248 edges, representing the interactions among these hub diagnostic TEX genes in all.

### Gene feature selection using LASSO, random forest, and SVM-RFE algorithms

**Fig. 6:**
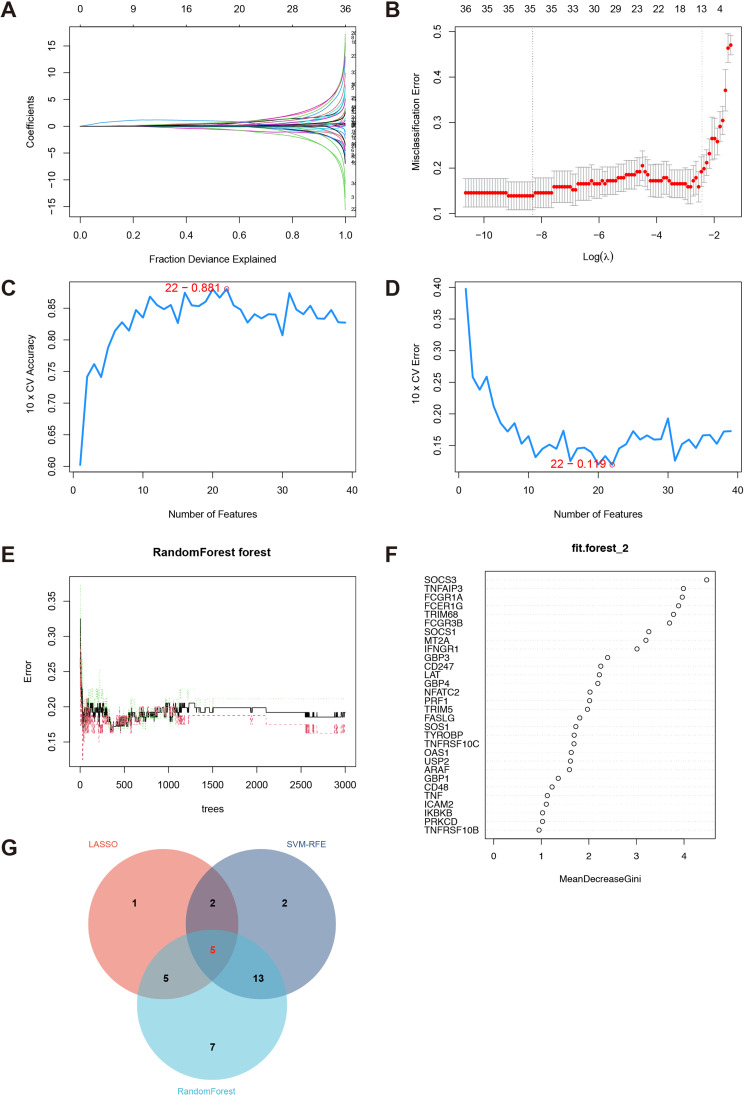
This figure consists of the following components: (**A**) Ten-fold cross-validation is a widely used technique for selecting optimum parameters in the LASSO model. Each curve is representative of a certain gene. (**B**) The graphic displays the coefficient profile of the LASSO regularization method. The presence of solid vertical lines in a graph signifies the representation of the standard error of the partial likelihood deviation. The vertical line denoted by dashes indicates the ideal value of lambda. The outcomes of the Support Vector Machine Recursive Feature Elimination (SVM-RFE) feature selection process are presented in this section, denoted as (**C**) and (**D**). (**E**) The present study investigates the correlation between the quantity of trees and the error rate within the context of the random forest algorithm. (**F**) The assessment of gene relative relevance. (**G**) A Venn diagram is presented to visually represent the common genes shared among the LASSO, random forest, and SVM-RFE algorithms

Three algorithms were utilized in the process of selecting feature genes from the TEX gene collection. The LASSO technique was employed, and a ten-fold cross-validation procedure was conducted to determine the optimal criteria for constructing the LASSO classifier. The criterion with the highest accuracy was selected as the minimal threshold. A comprehensive set of 13 genes of interest was identified, as visually depicted in Fig. 6A and B. The SVM-RFE technique demonstrated a reduction in classifier error when the number of features was limited to 22, as illustrated in Fig. 6C and D. The random forest technique identified a set of 30 genes that had comparatively higher relevance, as depicted in Fig. 6E and F. By means of cross-analysis, a set of five genes (CD48, CD247, FCER1G, TNFAIP3, and FCGR1A) was discovered as commonly featured by the LASSO, random forest, and SVM-RFE algorithms, as depicted in Fig. 6G.

### Derivation of T cell exhaustion-related features (construction of diagnostic score)

**Fig. 7:**
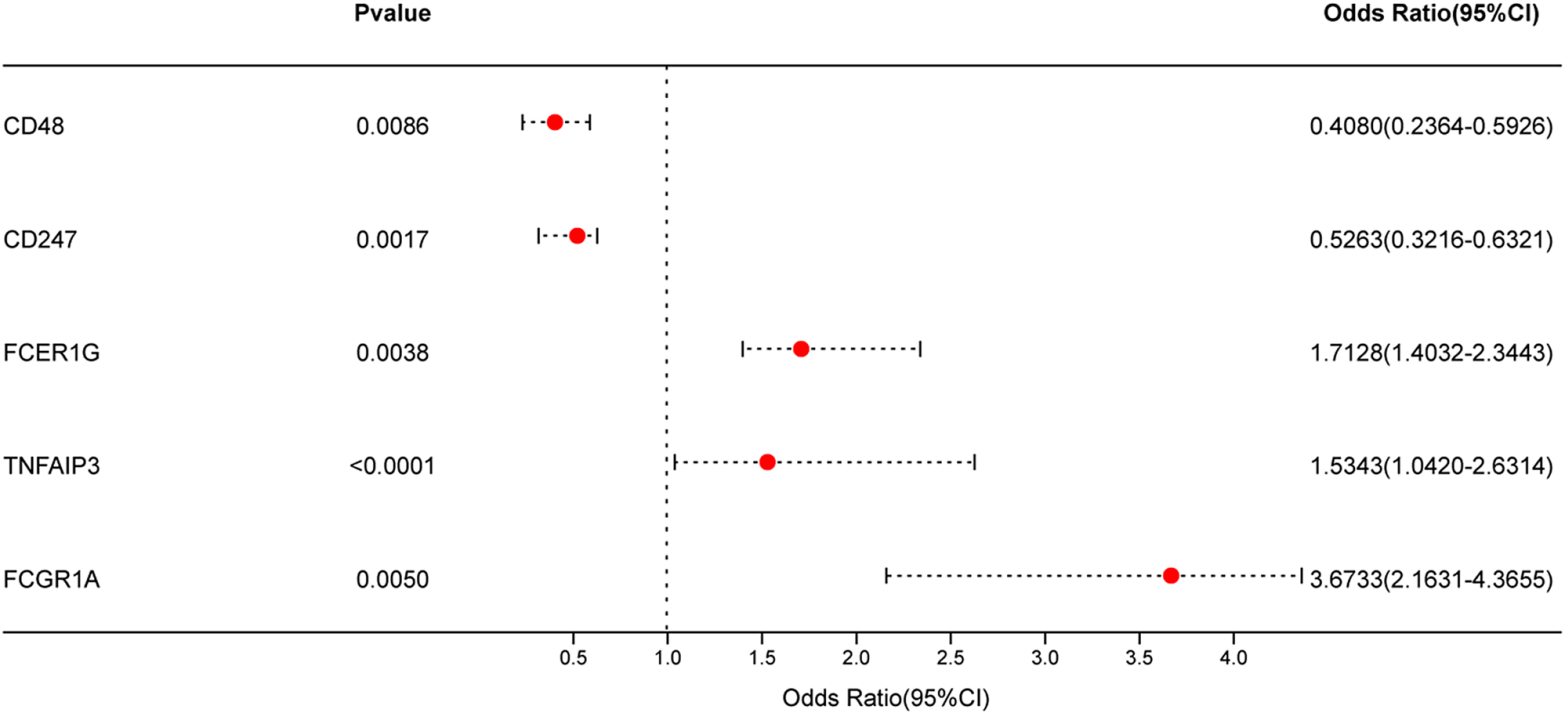
Forest plot of the 5 diagnostic TEX genes

Based on the expression data of the five hub TEX genes identified above, we performed multivariate logistic regression analysis to calculate the regression coefficients and expression levels of these hub TEX genes in the training datasets, thereby further constructing a diagnostic score. Figure 7 indicated the results of analysis above with forest plot, which also showed the direction of single gene effect. The detailed information could be all found in supplementary Table 2. Finally, the formula was as the following equation:

#### Validation and performance evaluation of diagnostic score

**Fig. 8:**
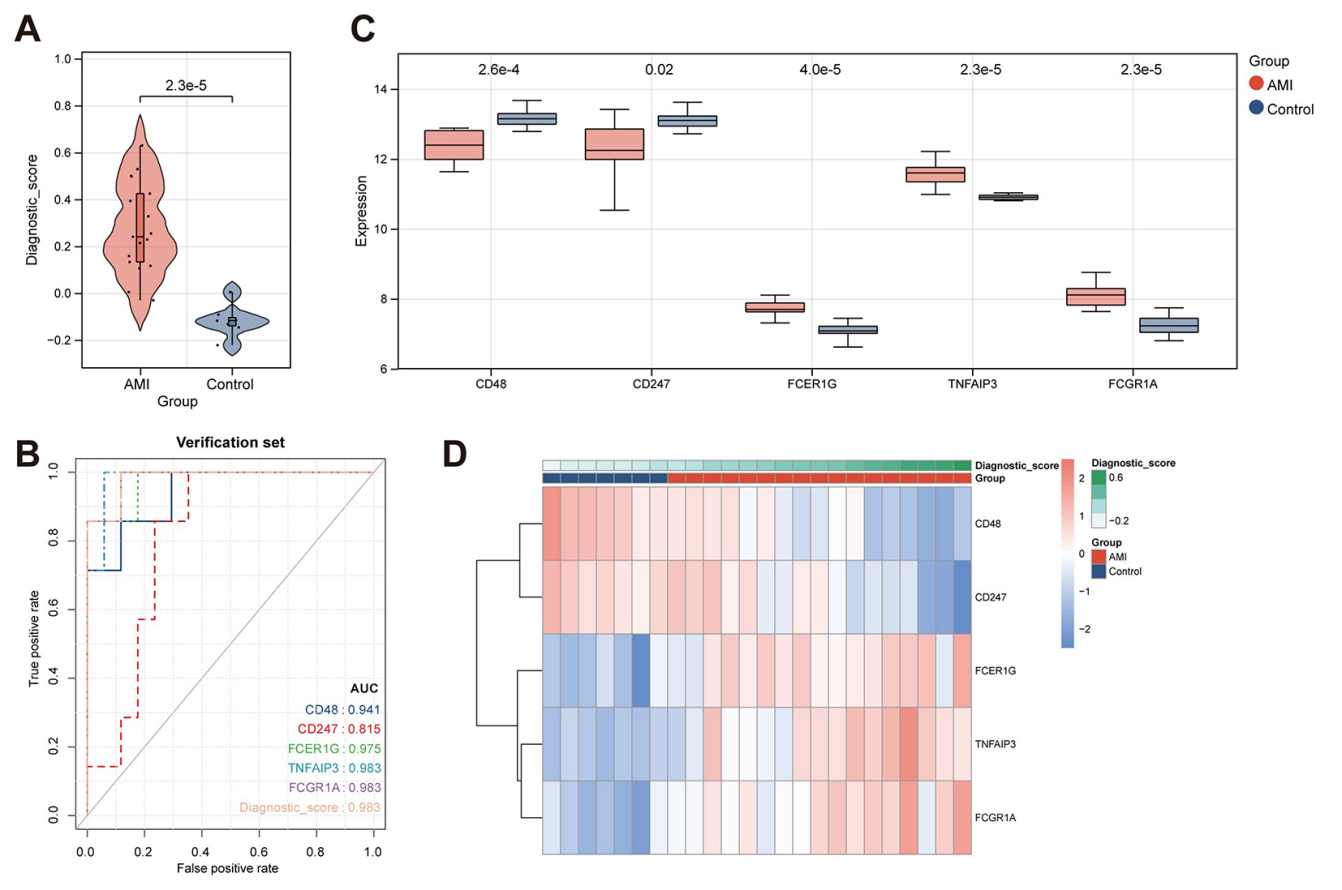
(**A**) Violin plot depicting the distribution of diagnostic scores for control and acute myocardial infarction (**AMI**) samples in the training set. (**B**) ROC curve analysis illustrating the predictive performance of the diagnostic scores. (**C**) Box plot displaying the expression levels of diagnostic genes. (**D**) Heatmap depicting the expression profiles of diagnostic genes

**Fig. 9:**
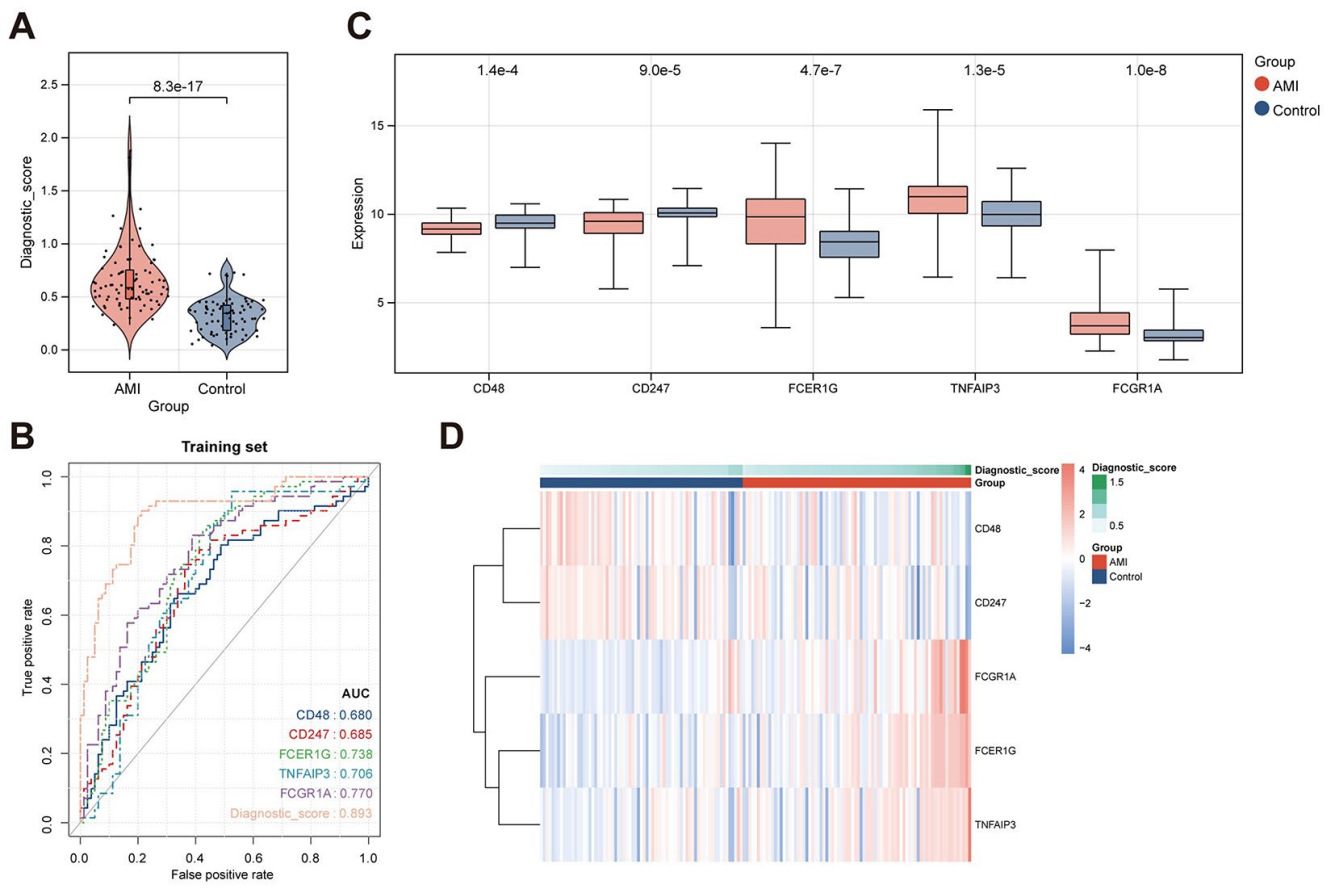
(**A**) Violin plot depicting the distribution of diagnostic scores for control and acute myocardial infarction (AMI) samples in validation set GSE60993. (**B**) ROC curve analysis illustrating the predictive performance of the diagnostic scores. (**C**) Box plot displaying the expression levels of diagnostic genes. (**D**) Heatmap depicting the expression profiles of diagnostic genes

Using the described methodology, we calculated the diagnostic scores for each sample in both the training set and the validation set (GSE60993). The results revealed that the diagnostic scores were able to effectively distinguish between control and acute myocardial infarction (AMI) samples, with AMI samples exhibiting significantly higher diagnostic scores compared to control samples (Fig. 8A). The ROC curve demonstrated excellent performance of the diagnostic scores in discriminating between control and AMI samples (Fig. 8B). Furthermore, as shown in Fig. 8C and D, the diagnostic genes exhibited significant differences between the control and AMI groups. Additionally, the external validation set further confirmed the prominent predictive value of the diagnostic scores (Fig. 9). Collectively, these findings demonstrate that the excellent predictive performance of our diagnostic model.

### Diagnostic nomogram and validation

**Fig. 10:**
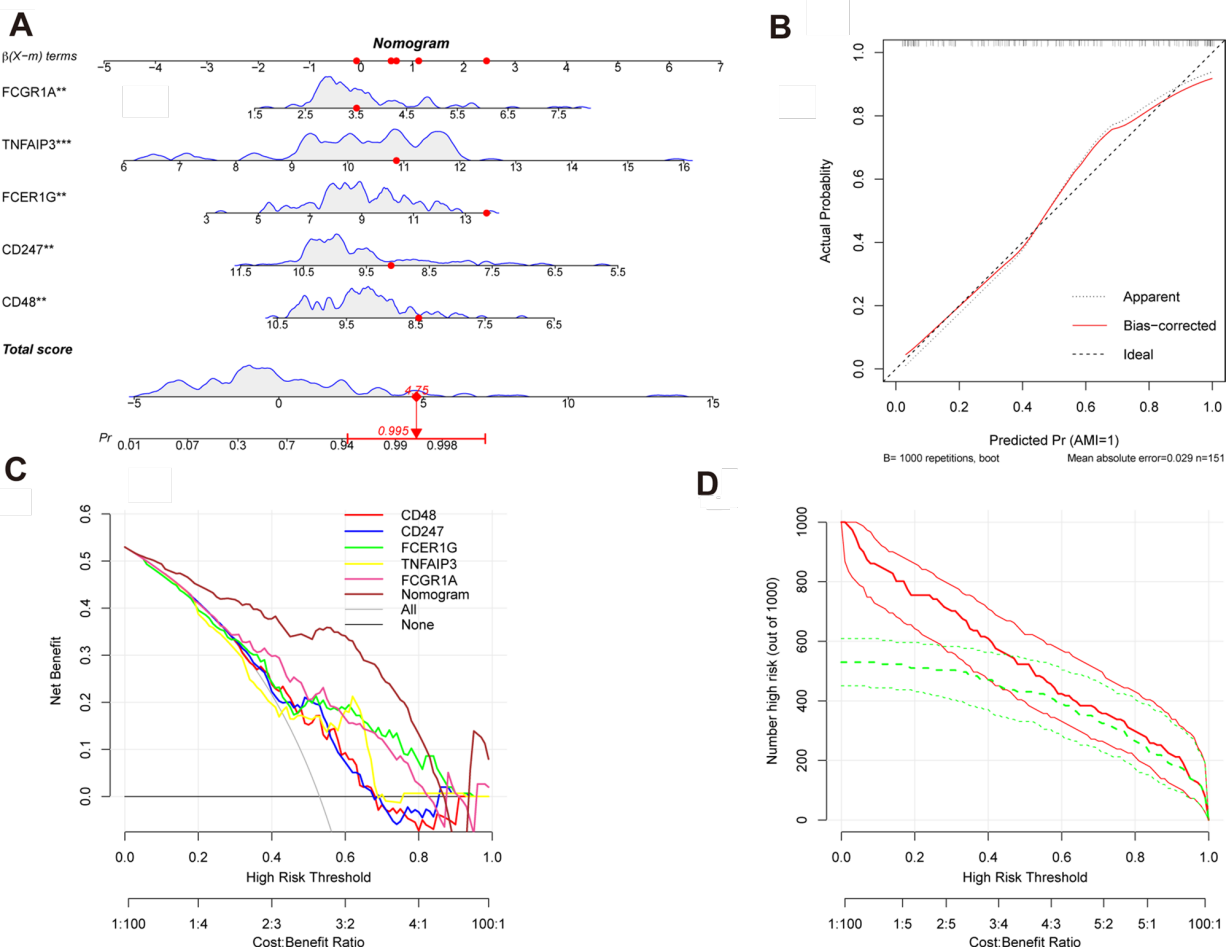
includes the following components: (**A**) Nomogram for diagnosing acute myocardial infarction (AMI). (**B**) Calibration curve used to assess the predictive ability of the nomogram. (**C**) Decision curve analysis (DCA) curve evaluating the clinical value of the nomogram. (**D**) Clinical impact curve based on the DCA curve assessing the clinical impact of the nomogram

To assess the diagnostic efficacy of the five chosen feature genes (CD48, CD247, FCER1G, TNFAIP3, and FCGR1A) for AMI, we integrated them into a nomogram (Fig. 10A). Following this, a calibration curve was employed to evaluate the predicted precision of the nomogram [31]. The calibration curve displayed a negligible divergence between the observed acute myocardial infarction (AMI) risk and the estimated risk, so showcasing a substantial level of predictive precision for the nomogram (Fig. 10B). The application of decision curve analysis (DCA) demonstrated that the nomogram curve exhibited improved performance compared to the grey line curve, therefore demonstrating a more favorable clinical utility of the nomogram in patients with acute myocardial infarction (AMI) (Fig. 10C). In order to enhance the comprehensibility of assessing the clinical significance of the nomogram, a clinical impact curve was generated utilizing the DCA curve [32]. The curve representing the number of high-risk individuals closely corresponded to the curve representing the number of high-risk individuals who experienced an event when the high-risk threshold was between 0.6 and 1. This observation indicates that the nomogram exhibited a strong predictive performance, as depicted in Fig. 10D.

#### Correlation analysis of diagnostic TEX genes with immune cells

**Fig. 11:**
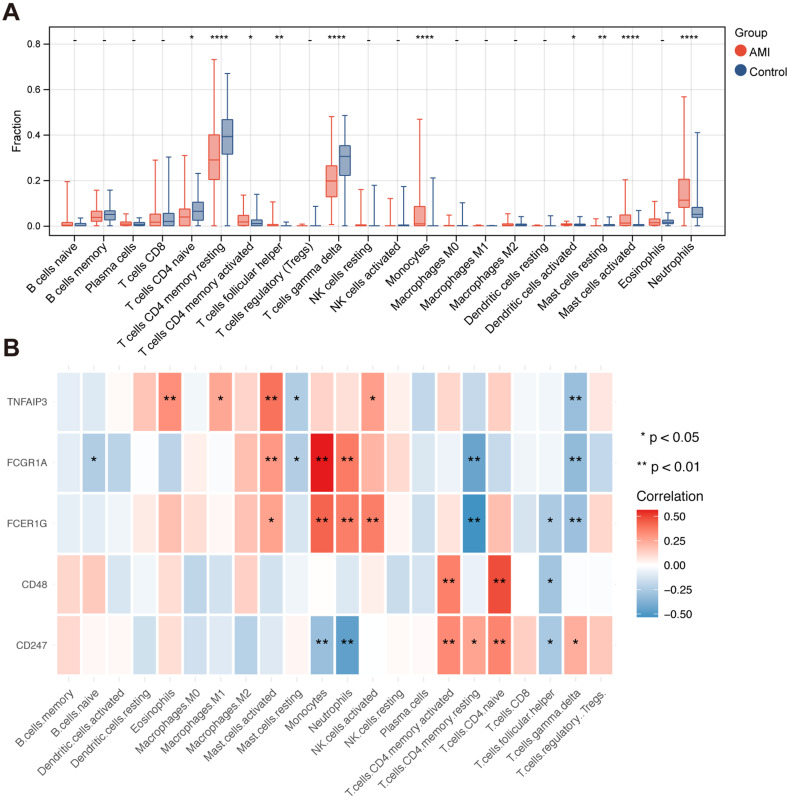
(**A**) Differential comparison of 22 immune cell types. (**B**) Correlation heatmap between 5 diagnostic TEX genes and 22 immune cell types

Utilizing the expression profile data, we utilized the CIBERSORT method to compute the proportions of immune cell types present in each sample, yielding proportions for a total of 22 distinct immune cell types. Following that, we conducted a comparative analysis of the disparities in immune cell proportions between the groups diagnosed with acute myocardial infarction (AMI) and the control group. By applying a significance threshold of *p* < 0.05, we have successfully identified ten distinct types of immune cells that display statistically significant distinctions. These types of immune cells are referred to as Differentiated Immune Cell types (DICs). The results of the intergroup comparison are depicted in Fig. 11A. In addition, a correlation analysis was conducted to examine the relationship between five diagnostic TEX genes and twenty-two immune cell types. The findings of this analysis are presented in Fig. 11B.

#### Gene set enrichment analysis

**Fig. 12:**
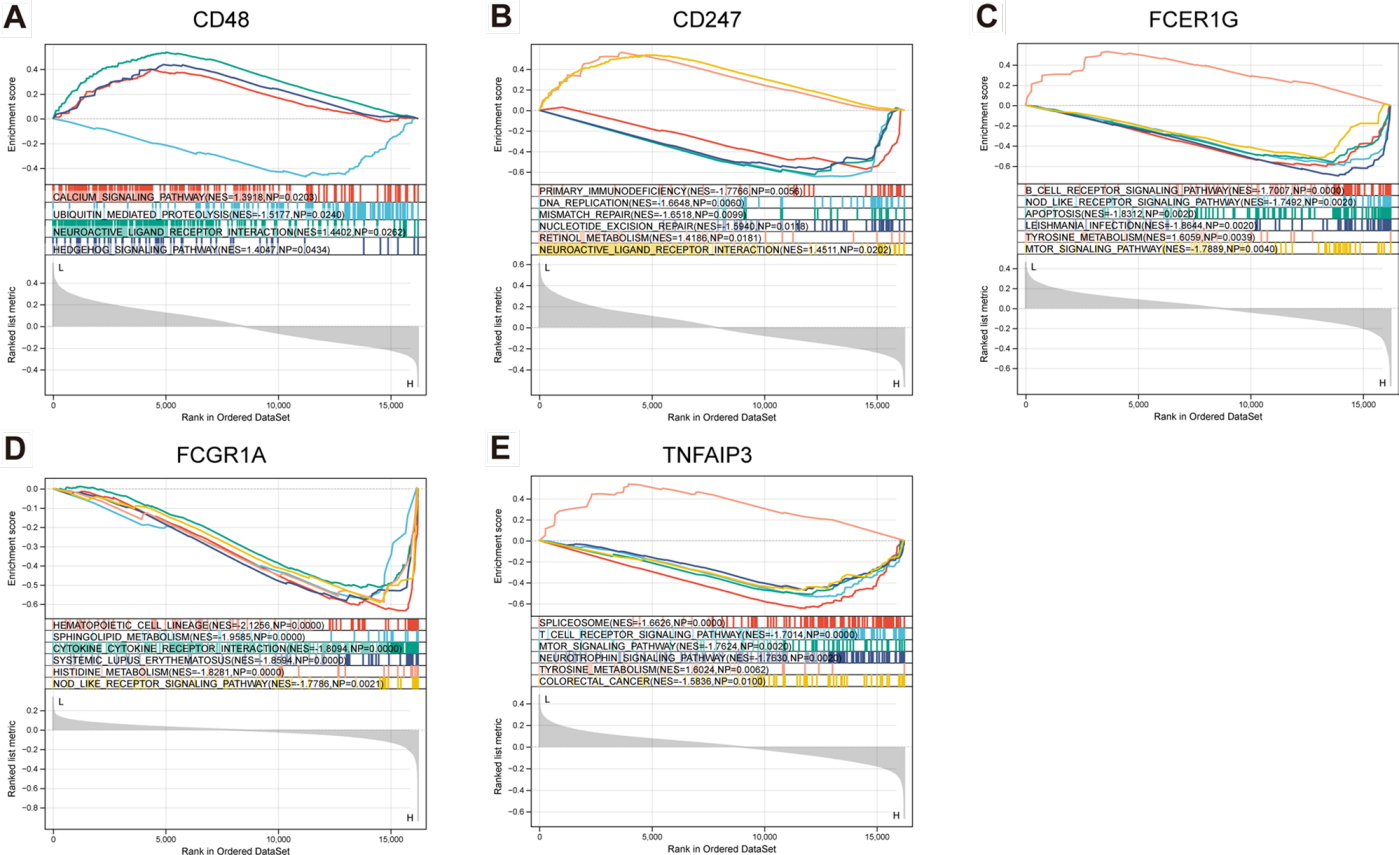
Enrichment of 5 diagnostic TEX-related KEGG gene set pathways

Utilizing the KEGG gene set, we conducted Gene Set Enrichment Analysis (GSEA) to ascertain noteworthy pathways. A significance criterion of p-value < 0.05 and an absolute NES (Normalized Enrichment Score) exceeding 1 [33] were employed for pathway identification. Following this, we conducted an analysis of the signaling pathways that are linked to the feature genes that were found. The primary findings are depicted in Fig. 12, while more comprehensive data may be accessed in the supplementary Table 3, specifically limited to the top six pathways.

#### Protein-protein interaction (PPI) analysis of diagnostic TEX genes

**Fig. 13:**
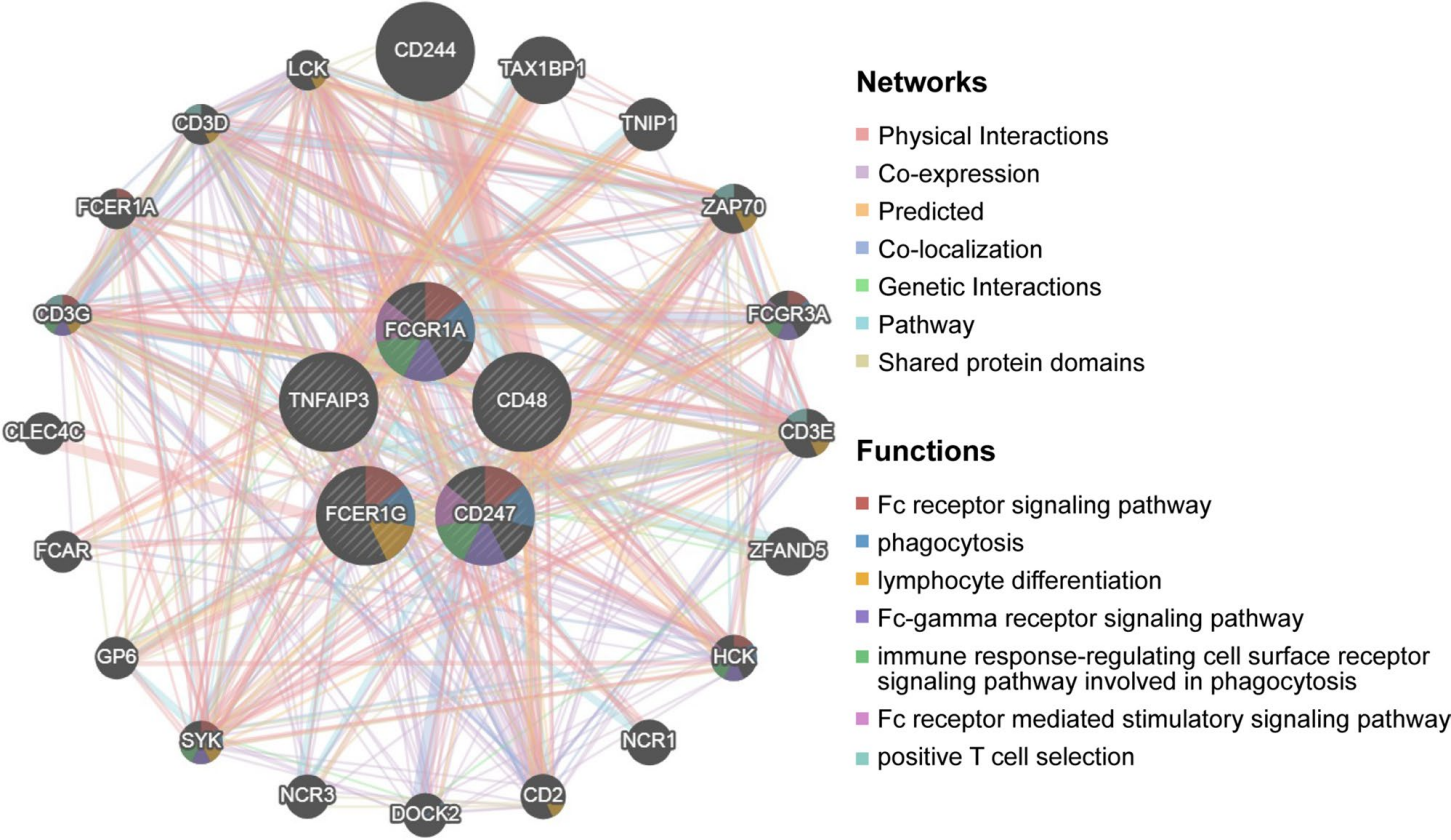
Gene-gene interaction network of 5 diagnostic TEX genes analyzed using the GeneMANIA database. The figure displays 20 neighboring genes with the most frequent changes. Each node represents a gene, and color of the nodes indicates to the potential functions of respective genes, which referred multiple biological activities

Using the GeneMANIA database, we performed functional analysis of the diagnostic TEX genes and constructed a gene-gene interaction network. The nodes in the network represent the 5 diagnostic TEX genes, which are surrounded by 20 nodes representing genes significantly associated with them (as shown in Fig. 13).

### Analysis of small molecule drug predictions

**Fig. 14:**
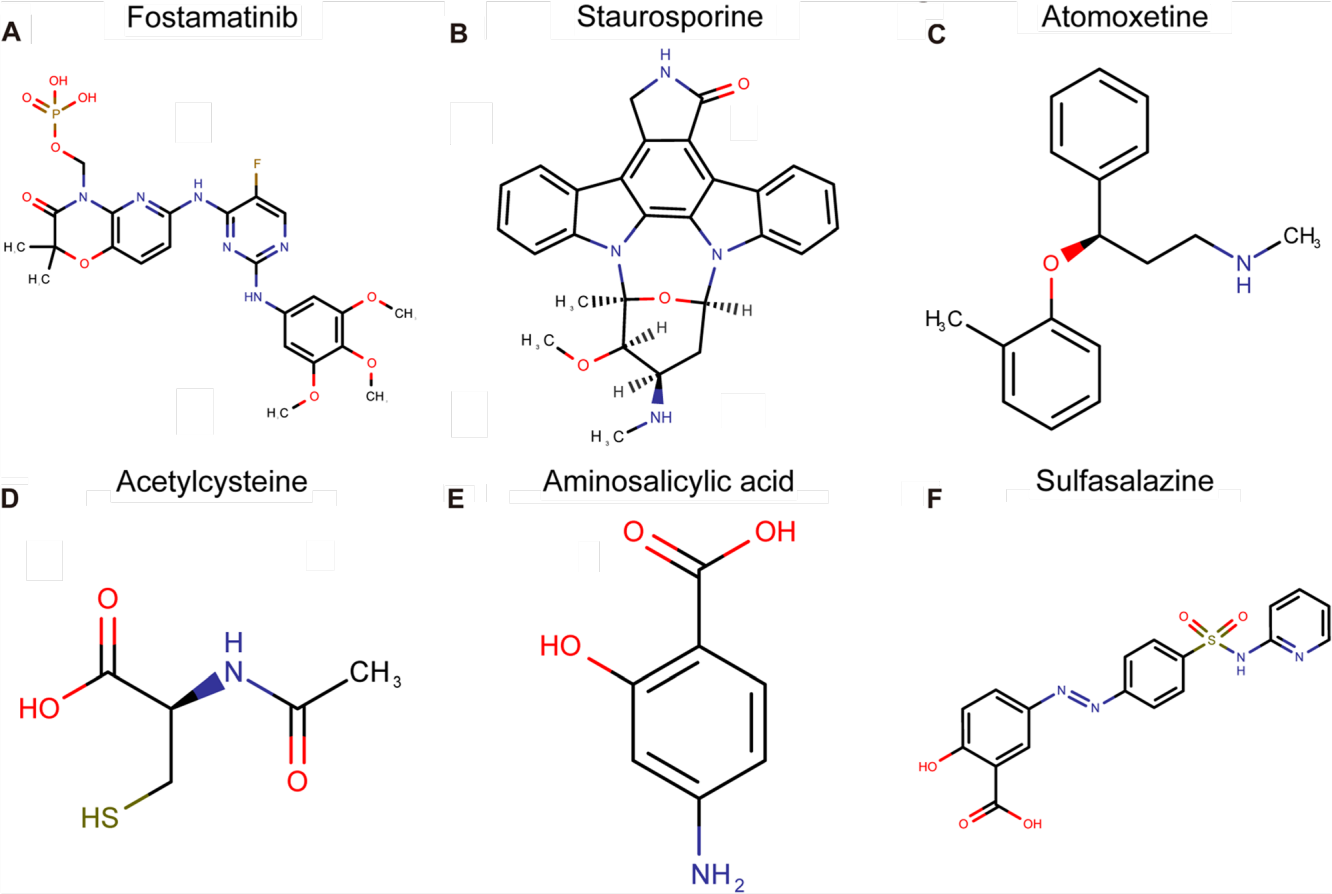
Molecular formulas of the 5 small molecule compounds

Based on the “DrugBank” database, we identified a set of small molecule candidate compounds that interact with five feature genes. The “DrugBank” database would automatically find the small molecule compounds with the tightest molecular affinity with five proteins we typed in corresponding to these five genes, of which the fundamental theory in these interactions is molecular docking. Detailed information can be found in the supplementary Table 4. Figure 14 displays the molecular formulas of 5 selected small molecule compounds.

## Discussion

In this study, we conducted a comprehensive analysis to investigate the role of T-cell exhaustion (TEX) in acute myocardial infarction (AMI) and its potential diagnostic implications. By integrating the transcriptomic profiling data and employing various computational approaches, we made significant findings that contribute to the understanding of TEX in AMI.

Initially, we effectively addressed batch effects and merged gene expression profiles obtained from AMI and control samples, thereby generating a consolidated dataset. The utilization of this dataset facilitated the execution of rigorous studies and enabled the derivation of dependable results pertaining to thromboembolism (TEX) in acute myocardial infarction (AMI). A list of 1725 genes with differential expression between acute myocardial infarction (AMI) and control samples was found using the process of differential expression analysis. Significantly, our study primarily examined the specific group of 39 genes that are linked to T-cell fatigue, hence providing insights into the potential role of TEX in the etiology of acute myocardial infarction (AMI). The results of this study indicate that TEX may have a significant impact on the regulation of the immunological response in the context of acute myocardial infarction (AMI). In order to have a deeper understanding of the functional implications associated with TEX-related genes, we conducted gene ontology (GO) and KEGG pathway enrichment analyses [34]. The findings of the study indicated a notable enrichment in many biological processes, cellular components, molecular activities, and KEGG signaling pathways [35]. The present enrichment study offers a comprehensive elucidation of the molecular mechanisms and pathways that potentially contribute to thromboembolic events (TEX) in the context of acute myocardial infarction (AMI).

Additionally, we constructed an interaction network of TEX genes, which unveiled potential molecular interactions and regulatory mechanisms underlying TEX in AMI. This network analysis not only provided valuable insights into the interconnectedness of TEX-related genes, but also further highlighted the key players within the whole network [36].

In order to create a diagnostic tool, machine learning methods were utilized to identify feature genes from the TEX gene collection. By means of a meticulous examination, we have successfully discovered the quintessential set of five genes (CD48, CD247, FCER1G, TNFAIP3, and FCGR1A) that are consistently observed across various algorithms. The genes were employed in the construction of a diagnostic score, which successfully differentiated between control and acute myocardial infarction (AMI) samples in both the training and validation datasets. The diagnostic model exhibited exceptional performance, as indicated by the examination of the receiver operating characteristic (ROC) curve and external validation. In addition, we investigated the association between the diagnostic TEX genes and the profiles of immune cells. Through the utilization of quantitative analysis to determine the relative proportions of several immune cell types, we have discerned notable variations between the AMI and control cohorts. Consequently, we have discovered a total of ten immune cell types that display statistically significant modifications. The correlation analysis conducted between the diagnostic TEX genes and immune cell types yielded significant insights into the interaction between TEX and immunological responses in acute myocardial infarction (AMI), particularly in respect to immune cell infiltration.

CD48 is a member of the SLAM (signalling lymphocyte activation molecule)-containing CD2 subfamily of the immunoglobulin superfamily (IgSF). On the surface of lymphocytes and other immune cells, dendritic cells, and endothelial cells, CD48 is involved in activation and differentiation pathways. The CD48 molecule facilitates interactions and contact between T cells and other immune cell types through its interaction with its ligand CD2 [37]. This mechanism is of paramount significance during the stimulation and growth phases of T cells. Moreover, the binding relationship between CD48 and CD2 regulates the entire immune response. This interaction enhances the cytotoxicity of cytotoxic T lymphocytes (CTL) on their target cells [38]. Acute myocardial infarction (AMI) and CD48 have not been linked conclusively by any studies to date. Given the close relationship between AMI and T-cell activation----for instance, the long-term immune response following a myocardial infarction, which may be associated with sustained T-cell activation and the formation of memory T cells----it is essential to emphasise CD48’s primary role in promoting T-cell activation. This may be one of the possible pathways connecting CD48 and AMI, but more research is necessary to confirm this hypothesis. CD247, also referred to as TCR (T cell receptor zeta chain) or CD3 (CD3 zeta chain), is a component of the T cell receptor (TCR) complex. Upon recognition and attachment of the antigenic peptide presented by antigen-presenting molecules, the TCR complex is activated [39]. In this process, the Immunoreceptor Tyrosine-based Activation Motifs (ITAMs) of CD247 are phosphorylated, promoting downstream signal transduction, which ultimately leads to T cell activation, proliferation, and cytokine secretion [40]. Similar to CD48, CD247 and Acute Myocardial Infarction (AMI) have not been conclusively linked by research. We hypothesize that the association between CD48 and AMI is also attributable to its influence on T cells.

FCER1G is the primary component of the immunoglobulin E (IgE) receptor and interleukin 3 (IL3) receptor complex, making it an essential molecule in allergic reactions. In addition to orchestrating the inflammatory pathway of mast cell-mediated allergic responses, it can also modulate a variety of other factors [41]. FCER1G is reported to share sequence variants with GP6 that influence the parameters associated with receptor-dependent thrombosis formation [42]. Previous research has implicated FCER1G in the recruitment of neutrophils, which serve as important cells in the AMI vascular inflammatory response and interact with macrophages. Due to its effect on plasma cluster activation and neutrophil recruitment, FCER1G may be associated with acute myocardial infarction [43].

TNFAIP3, also referred to as TNF- induced protein 3, is a cytokine-induced protein that inhibits cell apoptosis, activates NF-κB, and has a close relationship with inflammation. According to a study, TNFAIP3 gene-deficient rodents exhibited a substantially amplified inflammatory response relative to controls, concurrent with increased ubiquitination of RIP and TRAF6, all of which contributed to amplified NF-κB signals [44]. According to another study [45], TNFAIP3 inhibits the degradation of IκB within endothelial cells in environments with minimal shear stress, thereby providing negative feedback to NF-κB signals. It inhibits the migration of monocytes towards endothelial cells and protects against the proinflammatory conditions linked to the progression of atherosclerosis. TNFAIP3 can also be phosphorylated by IB kinase, which leads to ubiquitination and feedback inhibition of the NF-κB pathway. TNFAIP3 can inhibit the stimulation of inflammasomes comprised of NLRP3 within macrophages and related inflammatory responses, thereby stabilising atherosclerotic lesions [46].

There are several subtypes of Fc-R that can be classified as either activating or inhibiting in the human population. Fc-RI (CD64), Fc-RIIA (CD32a), Fc-RIIC (CD32c), Fc-RIIIA (CD16a), and Fc-RIIIB (CD16b) are involved in the activation of Fc receptors, whereas Fc-RIIB (CD32b) is the only inhibitory receptor. A 72-kDa transmembrane glycoprotein is encoded by the FCGR1A gene, also known as CD64. This glycoprotein possesses CD32 and CD16 receptors and functions as an Fc- receptor with high affinity [47]. CD64 expression on the surface of neutrophils is typically minimal. However, under certain pathological conditions, the presence of certain cytokines may swiftly increase CD64 expression on these cells. CD64 serves as a marker of neutrophil activation, and given the central role of neutrophils in acute myocardial infarction (AMI), there may be an association between CD64 and the occurrence and progression of AMI. Simultaneously, it has been demonstrated that neutrophils contribute to the exacerbation of atherosclerotic lesion instability in patients with acute myocardial infarction [48]. In general, Fc- receptors (Fc-R) and immunoglobulin G antibodies present in immune complexes are required for the activation of neutrophils in the context of lesions. According to existing research, there is evidence that the levels of unconnected Fc-RI on neutrophils decrease significantly in individuals with acute myocardial infarction. This finding highlights the potential contribution of aberrant Fc-RI expression to the instability of atherosclerotic plaques [49].

Gene set enrichment analysis (GSEA) revealed the significant pathways associated with the identified feature genes, further elucidating their functional implications in AMI [50]. Additionally, the protein-protein interaction (PPI) analysis provided a comprehensive understanding of the gene-gene interactions and potential regulatory mechanisms involved in TEX in AMI [51]. Lastly, we predicted a set of small molecule candidate compounds that interact with the diagnostic TEX genes, opening avenues for potential drug discovery and therapeutic interventions. In Fig. 14, we’ve presented the chemical conformation of five representative small molecule compounds with the strongest molecular binding force matched with five proteins. However, we didn’t find the suitable small molecule compounds for CD48. Figure 14 A showed the chemical conformation of Fostamatinib for CD247, which was a spleen tyrosine kinase inhibitor. Figure 14B was the chemical conformation of Cholesterol, herein, as the only candidate small molecule compound for FCER1G, which was apparently inadequate. Figure 14 C presented the chemical conformation of Atomoxetine for FCGR1A, which was a selective norepinephrine reuptake inhibitor (SNRI). Figure 14D was the chemical conformation of Acetylcysteine, which was generally a medication that can be used as a mucolytic in patients with certain lung conditions and as an antidote for acetaminophen overdose. Figure 14E showed the chemical conformation of Aminosalicylic acid for TNFAIP3, which is an aminosalicylate drug with the significant anti-inflammation function. Another candidate small molecule compound also for TNFAIP3----“Mesalazine” was the isomeride of “Aminosalicylic acid”. These small molecule compounds could possibly be the potential novel candidate drug for AMI in future. More experiments were demanded.

## Conclusion

In a word, our study provided a comprehensive understanding of TEX-related genes and their implications in AMI. The identification of diagnostic TEX genes, their interactions with immune cell profiles, as well as their association with the specific signaling pathways contributed to the understanding of AMI pathogenesis [52]. The diagnostic scoring system and nomogram based on TEX-related genes offered some promising tools for AMI risk stratification and personalized management. These findings have implications for the development of targeted therapies and precision medicine approaches in the field of AMI. Further research and validation are warranted to fully exploit the clinical potential of TEX-related genes in AMI diagnosis and treatment.

### Electronic supplementary material

Below is the link to the electronic supplementary material.

Supplementary Material 1

Supplementary Material 2

Supplementary Material 3

Supplementary Material 4

Supplementary Material 5

## Data Availability

The datasets applied in this study are readily accessible from the online repositories. Other data would be available from the corresponding author based on reasonable request.
