# Supplementary material for: Exploring T-cell exhaustion features in Acute myocardial infarction for a Novel Diagnostic model and new therapeutic targets by bio-informatics and machine learning
Source: BMC Cardiovasc Disord. 2024 May 24;24:272. doi: 10.1186/s12872-024-03907-x (PMC11118734; doi:10.1186/s12872-024-03907-x)
Supplement: Supplementary file 5 — Supplementary Material 5 [file 12872_2024_3907_MOESM5_ESM.doc]

**Supplementary table 1** differentially expressed genes in AMI and control groups

**Supplementary table 2** multivariate logistic regression analysis for 5 hub TEX genes

**Supplementary Table 3** signaling pathways for 5 feature genes

**Supplementary Table 4** Candidate drugs for 5 proteins
